# Urinary peptidomics and bioinformatics for the detection of diabetic kidney disease

**DOI:** 10.1038/s41598-020-58067-7

**Published:** 2020-01-27

**Authors:** Letícia de Almeida Brondani, Ariana Aguiar Soares, Mariana Recamonde-Mendoza, Angélica Dall’Agnol, Joíza Lins Camargo, Karina Mariante Monteiro, Sandra Pinho Silveiro

**Affiliations:** 1 Endocrine Division, Hospital de Clínicas de Porto Alegre (HCPA), Porto Alegre, RS, Brazil. Graduate Program in Medical Sciences: Endocrinology, Faculty of Medicine, Department of Internal Medicine, Universidade Federal do Rio Grande do Sul (UFRGS), Porto Alegre, RS Brazil; 2 Institute of Informatics, Universidade Federal do Rio Grande do Sul (UFRGS), Porto Alegre, RS Brazil; 3 Bioinformatics Core, Experimental Research Center, Hospital de Clínicas de Porto Alegre (HCPA), Porto Alegre, RS Brazil; 4 Laboratório de Genômica Estrutural e Funcional, Departamento de Biologia Molecular e Biotecnologia, Centro de Biotecnologia, Instituto de Biociências, Universidade Federal do Rio Grande do Sul (UFRGS), Porto Alegre, RS Brazil

**Keywords:** Computational biology and bioinformatics, Biomarkers, Endocrinology, Nephrology

## Abstract

The aim of this study was to establish a peptidomic profile based on LC-MS/MS and random forest (RF) algorithm to distinguish the urinary peptidomic scenario of type 2 diabetes mellitus (T2DM) patients with different stages of diabetic kidney disease (DKD). Urine from 60 T2DM patients was collected: 22 normal (stage A1), 18 moderately increased (stage A2) and 20 severely increased (stage A3) albuminuria. A total of 1080 naturally occurring peptides were detected, which resulted in the identification of a total of 100 proteins, irrespective of the patients’ renal status. The classification accuracy showed that the most severe DKD (A3) presented a distinct urinary peptidomic pattern. Estimates for peptide importance assessed during RF model training included multiple fragments of collagen and alpha-1 antitrypsin, previously associated to DKD. Proteasix tool predicted 48 proteases potentially involved in the generation of the 60 most important peptides identified in the urine of DM patients, including metallopeptidases, cathepsins, and calpains. Collectively, our study lightened some biomarkers possibly involved in the pathogenic mechanisms of DKD, suggesting that peptidomics is a valuable tool for identifying the molecular mechanisms underpinning the disease and thus novel therapeutic targets.

## Introduction

Diabetic kidney disease (DKD) is the main cause of end-stage renal disease in the United States^1^. In Europe, a quarter of patients that start renal replacement therapy has diabetes mellitus as the primary renal diagnosis^2^. The presence of reduced kidney function in patients with type 2 diabetes (T2DM) predominantly accounts for the observed increase in mortality^3^. In Brazil, the crude diabetes death rate increased 90%, while that of kidney disease due to diabetes more than doubled from 1990 to 2015^4^.

Diagnosis of DKD is based on the detection of elevated albuminuria and/or decreased glomerular filtration rate (GFR)^5^. The earliest putative diagnostic sign of diabetic renal damage is moderately elevated albuminuria^6,7^. However, substantial renal damage is already present at this stage. Furthermore, a non-albuminuric form of DKD, expressed by reduced GFR has been increasingly recognized, broadening the spectrum of the kidney involvement^8^. Reduced eGFR is generally estimated via creatinine-based equations^9^. Although demographic and clinical variables are included in the equation in an attempt to capture the variability in creatinine, the accuracy of equations is still disappointing^10,11^. Taken together, these evidence point that the current DKD clinical markers are nonspecific and late indicators of renal injury, suggesting the need for the search of early biomarkers, allowing interventions prior to established organ damage^12^. In addition, they can help the understanding of the pathogenesis of kidney disease and provide insight into novel therapeutic targets^13–17^.

The number of proteins identified associated with DKD has constantly increased, but none of the proposed urinary biomarkers has been shown to be superior to moderately increased albuminuria, the current gold standard for DKD diagnosis. The urinary peptidomic studies in DKD typically focus on the identification of biomarker panels instead of a single biomarker, and the multi-marker panels were able to demonstrate significant improvement in early DKD diagnosis and prognosis^15^. While large-scale studies of proteins are termed proteomics, the studies of naturally occurring peptides produced by endogenous protease activity are termed peptidomics^18^. A classifier composed of 273 urinary peptides (CKD273) associated with CKD, irrespective of the underlying etiology, has been previously described^19–21^. The predictive value of CKD273 in T1DM and T2DM patients has been demonstrated in its ability to anticipate the progression from normo- to macroalbuminuria long before the modification of albumin excretion rate^22^. Recently, CKD273 was shown to be independent of albuminuria and eGFR by enabled the identification of diabetic progressors to eGFR <60 mL/min/1.73 m^2^ in the absence of albuminuria^23^.

Using SELDI-MS analysis, a study showed a 12-peak signature predicting the development of DKD ten years prior to the increase in albumin to creatinine ratio^24^, while other study described a 4-peak pattern able to recognize T2DM patients with DKD^25^. A panel composed of 65 urinary peptide biomarkers, many of which fragments of type 1 collagen, was capable of distinguishing between diabetic patients without albuminuria from those with DKD, as well as predicting the progression toward overt DKD in patients with diabetes who had albuminuria over 3 years^15^. However, the lack of data reproducibility and the need of replication in populations with different genetic background and with different proteomic techniques pave the way for new studies.

Here, we reported the profile of naturally occurring urinary peptides from T2DM patients with different stages of DKD analyzed by LC-MS/MS. We used bioinformatics analyses to classify patients at different disease stages (A1, A2 or A3) according to their peptidomic profile and to identify peptides differentially represented between these disease stages. Bioinformatics tools were also used to investigate proteases possibly involved in the generation of these urinary peptides. Peptides from collagen, SERPING1 and SERPINA1, were identified as potential biomarkers to differentiate DKD stages. Possible roles for identified proteins and proteases in the pathogenic mechanisms of DKD were discussed.

## Results

### Characteristics of type 2 DM patients according to UAE

Table 1 depicts the characteristics of T2DM patients included in this study categorized by UAE according to the Kidney Disease: Improving Global Outcomes (KDIGO) guidelines. It was included 22 with normal (stage A1), 18 with moderately increased (stage A2) and 20 with severely increased albuminuria (stage A3) patients. eGFR was significantly decreased only in the A3 group as compared to A1 group. The three groups presented similar mean ages, diabetes duration, and HbA1c levels, as well as percentages of women and smokers. The presence of hypertension and the mean of systolic and diastolic blood pressure, BMI and waist were also similar among the 3 groups.

**Table 1:** Clinical and laboratory characteristics of type 2 DM patients with different stages of urinary albumin excretion (UAE) included in the study.

	A1 (Normal UAE)	A2 (Moderately Increased UAE)	A3 (Severely Increased UAE)	*p*-value
n	22	18	20	
Age (years)	62 ± 9	62 ± 13	62 ± 8	0.995
Diabetes duration (years)	19 (9–29)	19 (13–25)	16 (13–22)	0.780
Women (%)	64	44	45	0.584
ACEI/ARBs use (%)	89	87	75	0.474
Insulin use (%)	61	47	75	0.229
Metformin use (%)	89	88	65	0.136
Hypertension (%)	91	73	90	0.277
SBP (mmHg)	136 ± 19	127 ± 20	134 ± 22	0.394
DBP (mmHg)	76 ± 9	74 ± 15	78 ± 13	0.551
BMI (kg/m²)	31 ± 5	31 ± 5	32 ± 4	0.891
HbA1c (%)	8.8 ± 1.8	8.3 ± 2.4	8.8 ± 2.6	0.705
UAE (mg/L)	5 (3–9)	33 (22–72)	458 (292–752)	—
eGFR (mL/min/1.73 m²)	96 ± 17	86 ± 21	71 ± 33^a^	0.006

Values are expressed as means ± standard deviation, median (95% confidence interval) or percentage. ACEI: Angiotensin-converting-enzyme inhibitors, ARBs: Angiotensin receptor blockers, SBP: systolic blood pressure, DBP: diastolic blood pressure, eGFR: estimated glomerular filtration rate a Severely Increased vs. Normal (p=0.005).

### Urinary peptidomic analysis

**Figure 1:**
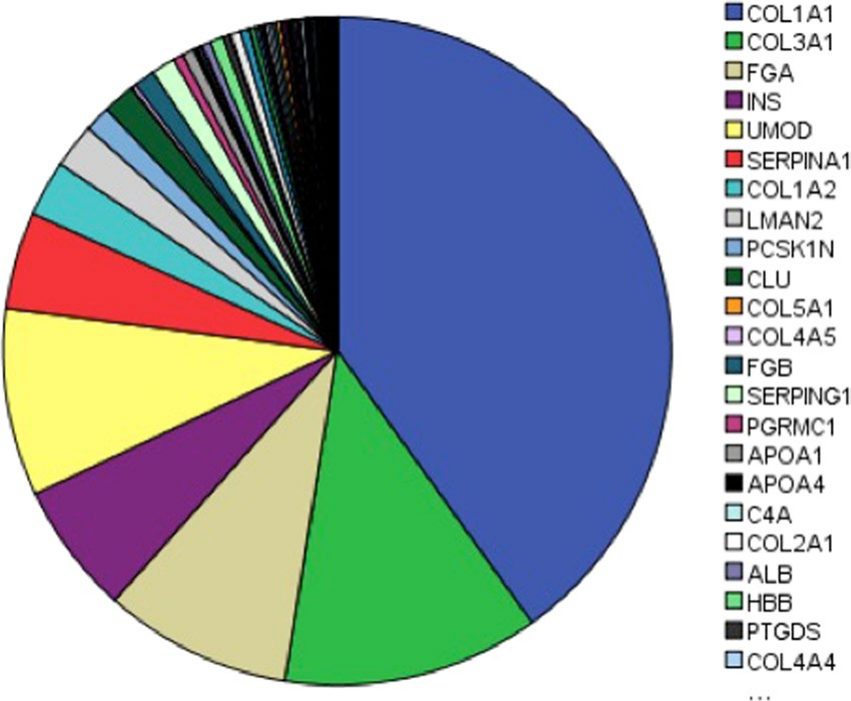
Protein-derived peptides found in the urine of T2DM patient’s irrespective of the DKD stage. Values are represented as spectral counts.

Naturally occurring peptides in the urine from patients of UAE stages A1, A2 and A3 were isolated by ultrafiltration and analyzed by LC-MS/MS. MS/MS data were searched against the UniProt human protein database for peptide and protein identification and a total of 1080 peptides were identified, which corresponded to a total of 100 proteins (Supplementary Table S1). Peptide abundance was assessed by label-free quantification using normalized spectral counts of individual peptides (Supplementary Table S1, S2 and S3**)**. The most abundant peptides detected in patients’ urine were from collagen-derived proteins (COL1A1 and COL3A1) (Fig. 1).

### Random forest model for prediction of disease stage

Based on the top 300 detected peptides among all samples, we trained a random forest (RF) model for classifying patients’ disease stage (A1, A2, or A3) according to their peptidomic profile. The detection rate for the filtered peptides varied between 100% (i.e., detected in all samples) and 5% (i.e., detected in 5% of samples). The confusion matrix for the tuned RF model, averaged over the 10 repetitions of the 5-fold cross-validation, is shown in Table 2. Among the 55 combinations of model’s parameters tested, the best performance was achieved with *ntree* = 500 and *mtry* = 21 (see Supplementary Fig. S1 for the complete results). An overall difficulty of the model in correctly classifying disease stage based on peptides quantification levels was observed. In particular, A1 patients were largely misclassified as A2, suggesting that peptidomic profiles of these two groups of patients may share similarities that turns the machine learning model training and classification more challenging. The average accuracy in the cross-validation process was 45.64% (±11.21%), with an AUC score of 0.619 (±0.106), and average sensitivity and specificity equal to 44.73% (±10.95%) and 72.58% (±5.60%), respectively. The same analysis was performed turning the data into a binary classification problem, with the two classes represented by A1 + A2 patients and A3 patients (Table 2). For this scenario, model tuning returned *ntree* = 500 and *mtry* = 9 as the best parameter combination (Supplementary Fig. S1). The average accuracy improved to 74.83%, with an AUC score of 0.746 (±0.1518), and average sensibility and specificity of 95.0% (±7.14%) and 34.5% (±21.9%), respectively. This improvement demonstrates that the main difficulty of the previous model is to differentiate between patients classified as A1 and A2. Due to this difficulty, further analyses were done using the binary variable (A1 + A2 vs. A3) as target value.

**Table 2:** Performance of the RF classifier estimated based on 10 repetitions of 5-fold cross-validation. Values are the average cell counts for the testing fold across all resamples.

		Reference		
		A1	A2	A3
Prediction	A1	2.4	2.1	1.3
	A2	1.5	0.9	0.6
	A3	0.5	0.5	2.1
		**A1** + **A2**		**A3**
	A1 + A2	7.6		2.6
	A3	0.4		1.4

A1: normal urinary albumin excretion (UAE), A2: moderately increased UAE, A3: severy increased UAE.

### Random Forest-based variable importance

**Figure 2:**
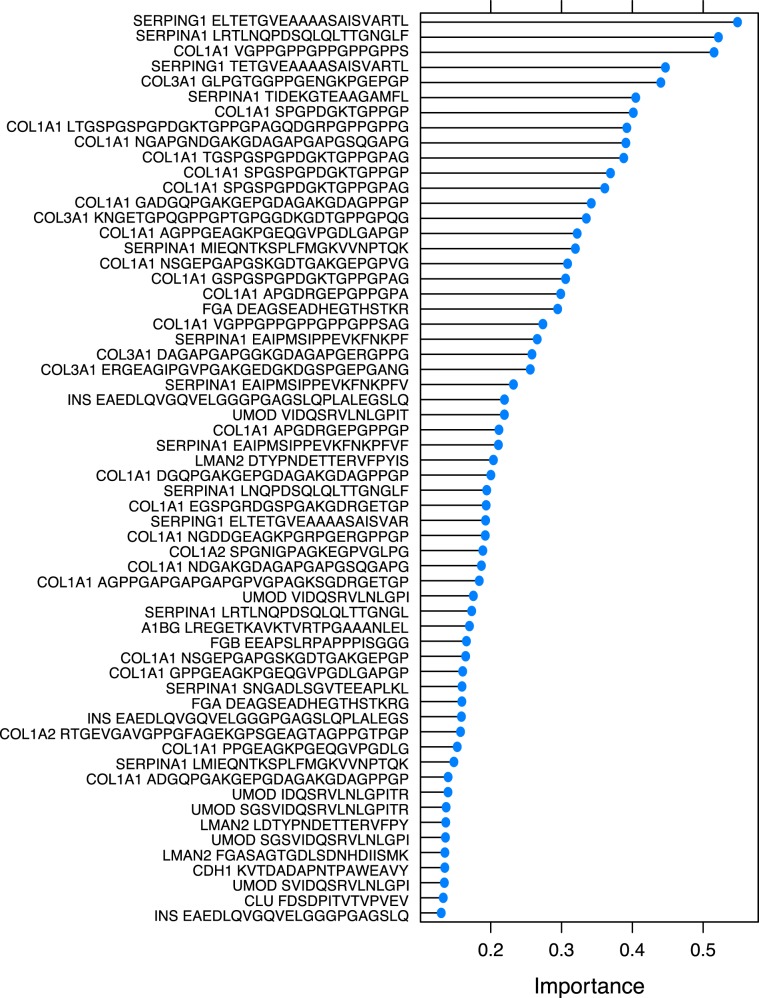
Variable importance estimated by the RF model for the top 60 predictors (peptides). Importance value (x-axis) represents average decrease in accuracy.

The RF analysis ranks variables (peptides) based on their predictive importance during model training, which may be useful to better understand how the variance observed in peptides quantification levels may be associated to patients’ DKD stages. Estimates for variable importance according to the average decrease in accuracy assessed during RF model training are shown in Fig. 2 (only the top 60 variables are shown. For the importance analysis of the 300 selected peptides used, see Supplementary File S1). The variables with the most predictive power included by ranking: multiple fragments of alpha-1 antitrypsin (SERPINA1; 10 fragments), type I collagen (COL1A1; 23 fragments), type III colagen (COL3A1; 4 fragments), C1 inhibitor (SERPING1; 3 fragments), but also other fragments as showed in Fig. 2.

**Figure 3:**
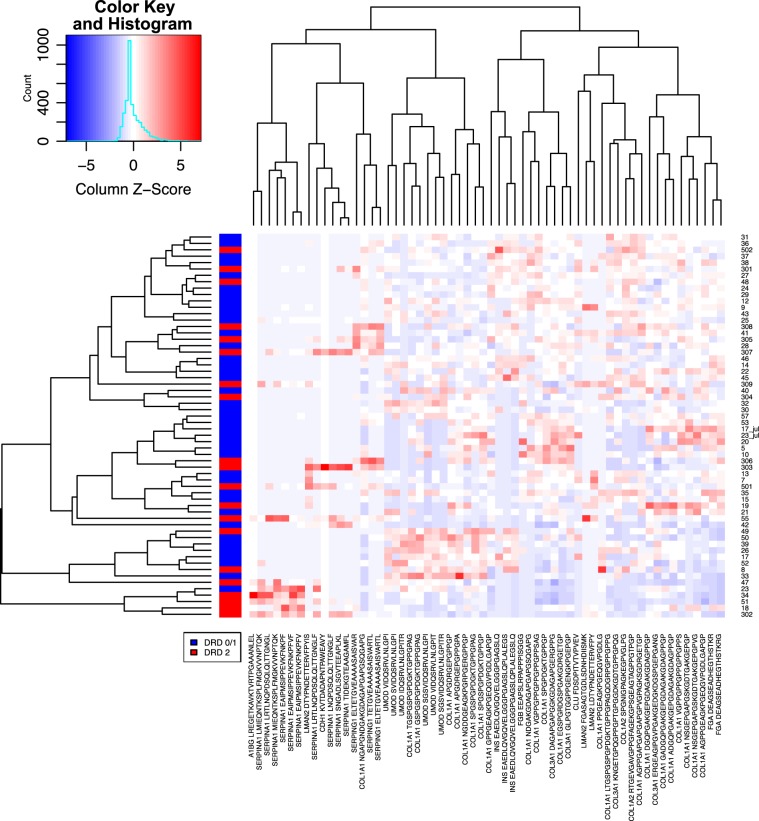
Heatmap visualization and hierarchical clustering performed for all samples considering the top 60 predictors according to Random Forests. Values are scaled across columns, generating column z-scores.

We performed hierarchical clustering of peptidomic profiles for all samples considering the 60 most important variables as assessed by the RF model (Fig. 3). Pearson correlation-based distance and complete linkage were adopted as parameters in the clustering algorithm. Results corroborate the difficulty in finding clear patterns associated to disease stage that may aid in the classification of patients’ diagnosis. Nonetheless, this analysis allows the visualization of singularities in peptides levels for subgroups of patients. For instance, four peptides associated to SERPINA1 show a particularly high concentration level for a subgroup of patients with advanced DKD (A3).

To further elucidate the expression pattern associated to the top predictors according to the RF model, we analyzed their fold change in the comparison between A1 + A2 patients and A3 patients. Of these peptides, 35 were found at lower and 25 at higher levels in A3 versus A1 and A2 groups (Table 3). Inspecting their statistical significance determined by the Mann-Whitney test, we observed that about half of peptides from the list (i.e., 29 out of 60) were significantly differentially expressed between groups (p < 0.05 and fold change <0.66 or fold change > 1.5), which are associated mainly to SERPINA1, COL1A1, and SERPING1. We note, however, that only 11 differentially expressed peptides showed statistically significant FDR value (FDR <0.05), probably due to the small sample size of our study (Supplementary File S2**)**. The values distribution for the top 60 predictors identified by the RF model according to patients’ disease stage (A1 and A2 or A3) are provided in Supplementary Fig. 2.

**Table 3:** Number of peptides with increased and decreased expression levels in A3 based on protein of origin (top 60 peptides according to RF model are presented). The numbers in parentheses correspond to peptides with statistically significant differential expression (p < 0.05 and fold change <0.66 or fold change >1.5), when present for a given protein.

Corresponding protein	Total peptides	Upregulated in A3	Downregulated in A3
COL1A1	23	6	17 (6)
SERPINA1	10	10 (10)	—
UMOD	6	—	6 (2)
COL3A1	4	—	4 (1)
INS	3	1	2
LMAN2	3	3 (2)	—
SERPING1	3	3 (3)	—
COL1A2	2	—	2
FGA	2	—	2 (1)
A1BG	1	1 (1)	—
CDH1	1	1 (1)	—
CLU	1	—	1 (1)
FGB	1	—	1 (1)

### Protease prediction

In silico analysis was performed in order to predict the proteases possibly involved in the generation of the 60 most important peptides of the present study. The Proteasix open-source peptide-centric tool was used for the analysis^26^. By using this bioinformatics approach it is possible to track back to the enzymes responsible for the generation of these peptides. The majority of human proteases have several protein targets and likewise, one peptide sequence could be cleaved by different proteases. The analysis yielded a list of 48 proteases putatively responsible for the generation of the 60 most important peptides (Supplementary Table S4**)**. From the 20 predicted proteases (Table 4), 9 proteases were already found deregulated in DKD patients: MMP-1, MMP-2, MMP-3, MMP-7, MMP-8, MMP-9, MMP-13, CTSD and CTSK^27^.

**Table 4:** List of predicted proteases by Proteasix tool.

	Protein name
CTSG	Cathepsin G
MMP2	Matrix metallopeptidase 2 (gelatinase A, 72kDa gelatinase, 72kDa type IV collagenase)
MEP1A	Meprin A, alpha (PABA peptide hydrolase)
CTSD	Cathepsin D
MMP8	Matrix metallopeptidase 8 (neutrophil collagenase)
MMP7	Matrix metallopeptidase 7 (matrilysin, uterine)
MMP13	Matrix metallopeptidase 13 (collagenase 3)
CTSK	Cathepsin K
CAPN1	Calpain 1, (mu/I) large subunit
CAPN2	Calpain 2, (m/II) large subunit
MMP3	Matrix metallopeptidase 3 (stromelysin 1, progelatinase)
MMP14	Matrix metallopeptidase 14 (membrane-inserted)
PGA3	Pepsinogen 3, group I (pepsinogen A)
MMP1	Matrix metallopeptidase 1 (interstitial collagenase)
MMP25	Matrix metallopeptidase 25
CTSE	Cathepsin E
MME	Membrane metallo-endopeptidase
ADAMTS4	ADAM metallopeptidase with thrombospondin type 1 motif, 4
MMP9	Matrix metallopeptidase 9 (gelatinase B, 92kDa gelatinase, 92kDa type IV collagenase)
TMPRSS7	Transmembrane protease, serine 7

## Discussion

The limitations of UAE and GFR as a predictive biomarkers of DKD pave the way for the search of new biomarkers using omics technologies^12,28^. Furthermore, since the DKD prevalence is continuously increasing, there is an urgency to identify the mechanisms underlying its pathogenesis^4^. In the present study, we investigated the urinary peptidomic pattern of T2DM patients with different stages of DKD. We used a more flexible data-driven approach instead of hypothesis-driven methods for the investigation of multiple peptides related to DKD stages. The use of bioinformatics tools is a well-suited alternative for conventional regression models that fails to include large numbers of covariates. Therefore, the random forest (RF) algorithm was used to rank variables based on their predictive importance during model training to better understand how the peptides levels variation may be correlated to patients’ diagnostic. In our study, considering the 3 stages of DKD, the RF approach achieved accuracy of below 70%, but accuracy raise when stage A1 and A2 were compared to stage A3, thus showing that the most severe DKD stage presented a distinct urinary peptidomic pattern when compared to two initials stages of disease. Next, the peptides were ranked in importance showing that the distinct pattern of A3 patients were due to an increased abundance of peptides from SERPINA1 and SERPING1 and a decreased abundance of collagen peptides.

Most of the differentialy expressed peptides found in the present study are in agreement with previous studies, despite the use of different proteomic methods, such as CE-MS^22,29,30^. Zurbig and collaborators^22^ analyzed urine peptides using a biomarker classifier for chronic kidney disease (CKD273) in a longitudinal cohort of diabetic patients. In this retrospective cohort, collagen fragments decreased before the increase of albumin excretion, showing a major role in the initiation of DKD^22^. The collagen-derived peptides were also observed relatively decreased in studies on CKD273 classifier for prognosis of CKD development, as well as in other proteomic analyses^29–31^. In the present study, we detected decreased levels of peptides from COL1A1 in urine of patients with the most severe DKD stage. These findings are also supported by previous hypothesis that urinary collagen fragments decrease might be an indicator of reduced collagen breakdown, resulting in fibrosis^32,33^. These peptides are likely the result of normal physiological turnover of the extracellular matrix. It has been assumed that diminished activity of matrix metalloproteinases may be responsible for the accumulation of proteins in the extracellular matrix and collagens that characterize the fibrotic kidney^34^. The collagen breakdown might be inhibited by the higher levels of tissue inhibitor of matrix metalloprotease type 1 observed in patients with renal disease^35^. Moreover, the accumulation of extracellular matrix as predominantly observed in DKD was shown to be associated with decreased excretion of several specific collagen fragments^32^.

Diabetes complications are intimately linked to inflammation, evidenced by the presence of high levels of plasma inflammatory markers such as high-sensitivity C-reactive protein^36^. Elevated acute-phase proteins may reflect the inflammation and activation of innate immune system during the course of DKD^37^. Related to inflammation, peptides from SERPING1 and SERPINA1 were found among the top 60 most important variables identified in the DKD severe stage (A3 stage). SERPINA1 is a serine protease inhibitor, which targets elastase as well as other proteases^38^. Neutrophil elastase degrades a range of substrates including elastin and other extracellular matrix proteins such as collagen, fibronectin, complement receptors and several growth factors^39^. Therefore, in DKD, the increased degradation of SERPINA1 would lead to activation of elastase and thus might contribute to accumulation of matrix molecules. In addition, SERPINA1 has been associated to non-protease inhibitory effects, including anti-inflammatory, anti-oxidant, and anti-apoptotic properties^40^. Although the exact mechanism remains unknown, previous studies also reported that peptide levels from SERPINA1 were increased in the kidney of microalbuminuria state, indicating a role for this protein in the pathogenesis of DKD^30,41,42^.

Increasing evidence points toward a role for the complement system in the pathogenesis of DKD^36^. *SERPING1* codes for a plasma protease C1 inhibitor that is involved in the regulation of the complement cascade^36^. In our study, SERPING1 peptides were increased in A3 patients’ urine. Likewise, these peptides were negatively correlated to eGFR and related to advanced CKD^43^. SERPING1 main function is the inhibition of the complement system, through the irreversible inactivation of C1r and C1s proteases of the classical pathway C1 complex, thus avoiding its spontaneous activation. On the other hand, activation of this pathway leads to the production of complement C3 convertase, which activates complement component C3, leading to generation of the opsonic C3b and eventually generation of the membrane attack complex, which lyses, damages, or activates target cells^36^. A disturbance of complement regulation might lead to different inflammatory actions that may be linked to the pathogenesis of DKD. Since we observed a higher SERPING1 degradation in A3 patients, it could be suggested that there is more activation of the complement system in the most severe DKD stage. Transcript levels of C3 and other complement components are increased in DKD glomeruli^44^. Additionally, mechanistic experiments performed on rodent diabetes models showed increased complement expression and activation correlated with DKD severity, whereas complement blockade improved outcomes in different nephropathy models^36,44^. Furthermore, patients and animals with DKD exhibited increased kidney C1q and C3 expression^45^. Based on these findings, and on the association of circulating immune complexes with diabetic renal injury, it is likely that complement system might play a functional role in DKD^45^.

The proteases regulation is necessary to maintain tissue homeostasis and its altered activity seems to be linked to the generation of peptides associated to DKD^27,46^. Bioinformatics peptide centric tools have been developed in order to track back to the proteases responsible for the generation of the urinary peptidome involved in DKD^26,47^. In the present study, we included DKD top 60 peptides in the Proteasix tool, yelding predicted proteases notably to the family of metalloproteinases, cathepsins and calpains. Supporting these findings, a recent study confirmed the altered activities and decreased expressions of some metalloproteinases in DKD at tissue levels^27^. The dysregulation of matrix metalloproteinases has been linked to renal fibrosis progression^48^ and DKD^49^, although the evidence is not always consistent^49,50^. MMP-2 and MMP-9 preferentially degrades collagen type IV^51^ – major component of tubular basement membrane (TBM) and linked to tubulointerstitial change and thickening of TBM^50,52^, hallmarks especifically related to DKD. Cathepsin D (CTSD), also verified in a previous study, is known to mediate inflammation^53^ and appeared increased in the human kidney tissue of DKD patients, especially in the areas of tubular damage^54^. From the same protease family, Cathepsin K (CTSK) seems to be involved in the DKD peptides generation and has been shown to be correlated with DKD progression and vascular endothelial dysfunction and deterioration of renal function^27^. Thus, the identification of protease dysregulation confirms the role of imbalance of collagen degradation, inflammation and fibrotic processes in DKD^27,47^.

Some factors may obscure the association between naturally occurring peptides and presence of kidney damage. Patients were burdened with coexisting diseases and a complex combination of drugs. These confounders might affect the urinary proteome and peptidome. The drugs known to reduce proteinuria such as angiotensin-converting-enzyme inhibitors and angiotensin receptor blockers certainly underestimated UAE levels. The study is crossectional, so the described association should be interpreted with caution and need further experimental verification. However, our analysis confirms the previously described findings in other populations and provided information that can be used for putative therapeutic targets and better understanding of DKD pathogenesis.

In conclusion, our LC-MS/MS analysis revealed that urinary peptide profile varies according to DKD severity, indicating a potential use for DKD risk stratification. The results were obtained by a sequence of bioinformatics approaches, first to rank the high important peptides in DKD context and second to allow the interpretation of integrated urinary peptidomics to the prediction of proteolytic events linked to DKD, emphasizing the differential regulation of inflammation and the complement system in DKD. Metalloproteases and cathepsins appear to be involved in the pathogenesis of DKD, highlighting the importance of further mechanistic studies. Due to the bioinformatics tools improvements, the information on urinary peptides should help to better define DKD on a molecular level and to identify specific therapeutic targets.

## Methods

### Clinical and laboratorial analyses of patients

A total of 60 patients with Type 2 DM (T2DM) from the Hospital de Clínicas de Porto Alegre (HCPA) had their urine samples consecutively collected using consistent standard operating procedures. All samples were collected as a spontaneously voided elimination and were stored immediately at −20 °C until analysis. The definition of T2DM was based on diagnosis of diabetes after the age of 40 years with no use of insulin during the first five years after diagnosis and no previous episodes of ketoacidosis. The exclusion criteria were malignancy and pregnancy. Informed written consent approved by responsible committee was obtained from all subjects, and ethical approval was obtained from the HCPA scientific committee.

A standard questionnaire was used to collect information on age, age at DM diagnosis, and drug treatment, and all patients underwent physical examination and laboratory evaluation. They were weighed unshod, wearing light outdoor clothes and their height was measured. Body mass index (BMI) was calculated as weight (kg)/height squared (meters). Blood pressure (BP) was measured twice using a digital sphygmomanometer (Omron) with the subject seated and a 5-minute rest between measurements. The means of both measurements were used to calculate systolic and diastolic BP. Hypertension was defined as BP levels of 140/90 mm Hg or higher, or if the patient was taking antihypertensive drugs^55^.

Type 2 DM patients were categorized according to urinary albumin excretion (UAE), and classified as normal if UAE <14 mg/L (KDIGO stage A1), moderately increased (stage A2) if UAE ranged from 14 to 174 mg/L and severely increased (stage A3) if UAE >174 mg/L^56,57^, confirmed in two out of three urine samples. UAE was determined by immunoturbidimetry (Sera-Pak immuno microalbuminuria; Bayer, Tarrytown, NY; mean intra- and interassay coefficients of variance of 4.5% and 7.6%, respectively)^55^.

GFR was estimated by CKD-EPI equation as recommended: GFR (mL/min/1.73 m^2^) = 141 × min(Scr/k,1)^a^ × max(Scr/k,1)-^1.209^ × 0.993^Age^ × 1.018 (if female) × 1.159 (if black), where Scr is serum creatinine, k is 0.7 for females and 0.9 for males, a is −0.329 for females and –0.411 for males, min indicates the minimum of Scr ⁄ k or 1, and max indicates the maximum of Scr ⁄ k or 1^55^.

A serum sample was taken after 12 hours of fasting for laboratory analyses. Creatinine levels were determined using a traceable Jaffe reaction kit; glycated hemoglobin (HbA1c) was quantified using an ion-exchange HPLC procedure (Merck-Hitachi L-9100 GhB Analyzer; Merck, Darmstadt, Germany; reference range: 4.7%–6.0%).

### Urinary peptide isolation

A 1.2-ml aliquot of urine was thawed immediately prior to use and diluted with 0.6 ml of an aqueous solution supplemented with 3 M urea, 15 mM NH_4_OH, and 0.03% (w/v) SDS. To remove proteins of high molecular mass, the sample was filtered using Centrisart ultrafiltration devices (20-kDa molecular mass cutoff; Sartorius, Goettingen, Germany) at 2,000 g for 10 min at 18 °C^19^. Subsequently, 1.2 ml of filtrate was applied onto a PD-10 desalting column (GE Healthcare) equilibrated with 0.01% NH_4_OH to remove urea, electrolytes, and salts. Additionally, 1.3 ml of equilibration buffer was applied to the filter bed as a first step and allowed to wash out by gravity flow, improving the yield of naturally occurring peptides. Next, 2 ml of equilibration buffer was applied to the PD-10 column. The flow-through was collected, lyophilized, and stored at −20 °C until further use^19^.

### Liquid chromatography-tandem mass spectrometry (LC-MS/MS) analysis and database searching

Urinary peptides were analyzed by liquid chromatography-tandem mass spectrometry (LC-MS/MS) using a Waters nanoACQUITY UPLC system coupled to a Q-TOF Ultima API mass spectrometer (Waters, Milford, MA, USA), as described previously^58^. The peptides were eluted from the reverse-phase column toward the mass spectrometer at a flow rate of 200 nl/min with a linear gradient of acetonitrile in 0.1% formic acid (2–90%, 60 min). The MS survey scan was set to 1 s (0.1 s interscan time) and recorded from 100 to 2000 m/z. MS/MS scans were acquired from 50 to 2000 m/z, and scan and interscan rates were set as for MS. The samples were run in DDA mode where each full MS scan was followed by MS/MS acquisition using collision-induced dissociation of the three most intense ions from the MS scan. Each sample was run in duplicates (technical replicates). MS/MS raw data were processed using ProteinLynx Global Server software (Waters) and peak lists were exported in the mascot generic (.mgf) format. Peptides and protein identifications were performed with the MASCOT search engine (Matrix Science, London, UK; version 2.3.0) against UniProt/Swiss-Prot human protein database^59^. Database search was performed with the following parameters: oxidation of methionine (M), proline (P) and lysine (K) as variable modifications, without any enzyme specificity and maximal missed cleavage of 0. A mass tolerance of 0.1 Da for precursor and fragment ions was set. Ion type was set as monoisotopic, and peptide charges 2+, 3+ and 4+ were taken into account. Protein and peptide identifications were validated by Scaffold (Proteome Software Inc., version 4.4.1.1) analysis. MASCOT *.dat files were loaded on Scaffold and peptide identifications were accepted if they could be established at >95% of probability as assigned by the Peptide Prophet algorithm^60^ and protein identifications were accepted if they could be established at >99% of probability as assigned by the Protein Prophet algorithm^61^ and contained at least 2 identified peptides. The false discovery rate (FDR, Decoy) was <1% for proteins and peptides. The spectral counts were calculated for each peptide using Scaffold and normalized following default settings in which spectral counts are multiplied by the average across all samples and divided by the total number of spectral counts within each sample^62^.

### Estimating variables importance with Random Forests

Random forest (RF) is an algorithm for classification that uses an ensemble of decision trees, each of which is built using a bootstrap sample of the data^63^. In addition, RF adopts random variable selection during trees growth that when combined to the bootstrap aggregation (i.e., bagging) procedure tends to generate a collection of weakly correlated individual trees. As a result, the ensemble-based decision has a great potential to achieve low bias and low variance in predictions, leading to improved predictive performance in relation to individual trees. RF has demonstrated excellent performance in genomic classification tasks, comparable to other state-of-the-art methods such as Support Vector Machines (SVMs)^64^. However, its ability to evaluate and rank variables based on their predictive importance during model training makes it particularly well-suited to better understand how the variance observed in peptides quantification levels may be associated to patients’ diagnostic.

For this purpose, we trained a RF classifier using as training data the quantification of peptides for the 60 subjects. Common data pre-processing steps, such as removing highly correlated variables or variables with near zero variance across samples, were not applied due to the sparse nature of data and to the expected correlation among peptides related to the same protein. Since most peptides have over 50% missing values, only the top 300 detected peptides (i.e., in decreasing order of detection rate) were considered as model’s variables. The number of trees in the ensemble (*ntree*) and the number of variables randomly sampled as candidates for node splitting during the tree growing process (*mtry*) were tunned, testing all possible combinations of *ntree* = [500, 1000, 1500, 2000, 2500] and *mtry* = [7, 9, 11, 13, 15, 17, 19, 21, 23, 25, 27]. Mtry values were chosen by defining an interval of −10 and +10 around the default suggested value (i.e., the square root of the total number of variables, which would be approximately 17 for our data), in steps of two. Model performance was assessed using the sensitivity, specificity, and accuracy computed based on the confusion matrix, as well as the area under the Receiver Operating Characteristic (ROC) curve (i.e., AUC score). Relative importance was estimated for each variable during model training as the mean decrease in accuracy observed after permuting the predictor’s values. Analyses were performed with R package caret v.6.80.

### Protease prediction

The open-source tool for protease prediction – Proteasix (www.proteasix.org)^26^ was used for the analysis to link naturally occurring peptides in urine to the proteases potentially involved in their generation. Proteasix uses information about naturally occurring peptides i.e. corresponding protein UniProt ID and start and stop amino acid position to predict potential cleaving proteases^27^. Proteasix retrieves information about cleavage sites (CS) from protease databases (MEROPS, BRENDA) considering also cleavage site restrictions (from ENZYME database)^27^. A list of predicted proteases is generated as a result of the analysis.

### Statistical analysis

One-way ANOVA and χ2 tests were performed when appropriate. Statistical significance was assumed at p < 0.05. Peptide differential expression was assessed with the Mann-Whitney test and P-values were adjusted for multiple testing using the Benjamini-Hochberg method (FDR, false discovery rate). Peptides were considered differentially expressed among groups based on p < 0.05 and ratio higher than 1.5 (fold change higher than 1.5 or lower than 0.66)^27^. Statistical analyses were performed using SPSS version 18.0 (SPSS, Chicago, IL, USA) and R statistical language.

## Supplementary information

Supplementary information.

Supplementary information2.

Supplementary information3.

Supplementary information4.

Supplementary information5.

Supplementary information6.

Supplementary information7.

Supplementary information8.

## Data Availability

All data generated or analyzed during this study are included in this published article (and its Supplementary Information files). As described above, all methods were carried out in accordance with relevant guidelines and regulations.

## References

[1] Narres M (2016). The Incidence of End-Stage Renal Disease in the Diabetic (Compared to the Non-Diabetic) Population: A Systematic Review. PLoS one.

[2] Kramer A (2018). The European Renal Association - European Dialysis and Transplant Association (ERA-EDTA) Registry Annual Report 2015: a summary. Clin. Kidney J..

[3] Afkarian M (2013). Kidney disease and increased mortality risk in type 2 diabetes. J. Am. Soc. Nephrology: JASN.

[4] Duncan BB (2017). The burden of diabetes and hyperglycemia in Brazil-past and present: findings from the Global Burden of Disease Study 2015. Diabetology Metab. syndrome.

[5] Barrett EJ (2017). Diabetic Microvascular Disease: An Endocrine Society Scientific Statement. J. Clin. Endocrinol. Metab..

[6] Mazzucco G (2002). Different patterns of renal damage in type 2 diabetes mellitus: a multicentric study on 393 biopsies. Am. J. kidney diseases: Off. J. Natl Kidney Found..

[7] Jun M (2018). Changes in Albuminuria and the Risk of Major Clinical Outcomes in Diabetes: Results From ADVANCE-ON. Diabetes care.

[8] American Diabetes Association (2018). 10. Microvascular Complications and Foot Care: Standards of Medical Care in Diabetes-2018. Diabetes care.

[9] Levey AS (2009). A new equation to estimate glomerular filtration rate. Ann. Intern. Med..

[10] Silveiro SP (2011). Chronic Kidney Disease Epidemiology Collaboration (CKD-EPI) equation pronouncedly underestimates glomerular filtration rate in type 2 diabetes. Diabetes care.

[11] Gentile G, Remuzzi G (2016). Novel Biomarkers for Renal Diseases? None for the Moment (but One). J. Biomol. Screen..

[12] Pena MJ (2015). Prognostic clinical and molecular biomarkers of renal disease in type 2 diabetes. Nephrology Dialysis Transplant..

[13] Pena MJ, Mischak H, Heerspink HJ (2016). Proteomics for prediction of disease progression and response to therapy in diabetic kidney disease. Diabetologia.

[14] Mischak H (2015). Pro: Urine proteomics as a liquid kidney biopsy: No more kidney punctures!. Nephrology Dialysis Transplant..

[15] Rossing K (2008). Urinary proteomics in diabetes and CKD. J. Am. Soc. Nephrology: JASN.

[16] Albalat A, Mischak H, Mullen W (2011). Clinical application of urinary proteomics/peptidomics. Expert. Rev. Proteom..

[17] Ben Ameur R (2010). Proteomic approaches for discovering biomarkers of diabetic nephropathy. Nephrology, dialysis, transplantation: Off. Publ. Eur. Dialysis Transpl. Assoc. - Eur. Ren. Assoc..

[18] Van JA, Scholey JW, Konvalinka A (2017). Insights into Diabetic Kidney Disease Using Urinary Proteomics and Bioinformatics. J. Am. Soc. Nephrology: JASN.

[19] Good DM (2010). Naturally occurring human urinary peptides for use in diagnosis of chronic kidney disease. Mol. Cell. proteomics: MCP.

[20] Pontillo C (2017). A urinary proteome-based classifier for the early detection of decline in glomerular filtration. Nephrology, dialysis, transplantation: Off. Publ. Eur. Dialysis Transpl. Assoc. - Eur. Ren. Assoc..

[21] Pontillo C (2017). Prediction of Chronic Kidney Disease Stage 3 by CKD273, a Urinary Proteomic Biomarker. Kidney Int. Rep..

[22] Zurbig P (2012). Urinary proteomics for early diagnosis in diabetic nephropathy. Diabetes.

[23] Zurbig P, Mischak H, Menne J, Haller H (2019). CKD273 Enables Efficient Prediction of Diabetic Nephropathy in Nonalbuminuric Patients. Diabetes care.

[24] Otu HH (2007). Prediction of diabetic nephropathy using urine proteomic profiling 10 years prior to development of nephropathy. Diabetes care.

[25] Wu J, Chen YD, Yu JK, Shi XL, Gu W (2011). Analysis of urinary proteomic patterns for type 2 diabetic nephropathy by ProteinChip. Diabetes Res. Clin. Pract..

[26] Klein J (2013). Proteasix: a tool for automated and large-scale prediction of proteases involved in naturally occurring peptide generation. Proteom..

[27] Krochmal M (2017). Urinary peptidomics analysis reveals proteases involved in diabetic nephropathy. Sci. Rep..

[28] Lin CH, Chang YC, Chuang LM (2016). Early detection of diabetic kidney disease: Present limitations and future perspectives. World J. Diabetes.

[29] Lewandowicz A (2015). Changes in urine proteome accompanying diabetic nephropathy progression. Polskie Archiwum Medycyny Wewnetrznej.

[30] Alkhalaf A (2010). Multicentric validation of proteomic biomarkers in urine specific for diabetic nephropathy. PLoS one.

[31] Araki S (2014). Novel biomarkers for diabetic nephropathy. Rinsho byori. Japanese J. Clin. Pathol..

[32] Rossing K (2008). The urinary proteome in diabetes and diabetes-associated complications: New ways to assess disease progression and evaluate therapy. Proteom. - Clin. Appl..

[33] Andersen S, Mischak H, Zurbig P, Parving HH, Rossing P (2010). Urinary proteome analysis enables assessment of renoprotective treatment in type 2 diabetic patients with microalbuminuria. BMC nephrology.

[34] Cheng S, Pollock AS, Mahimkar R, Olson JL, Lovett DH (2006). Matrix metalloproteinase 2 and basement membrane integrity: a unifying mechanism for progressive renal injury. FASEB journal: Off. Publ. Federation Am. Societies Exp. Biol..

[35] Horstrup JH (2002). Elevation of serum and urine levels of TIMP-1 and tenascin in patients with renal disease. Nephrology, dialysis, transplantation: Off. Publ. Eur. Dialysis Transpl. Assoc. - Eur. Ren. Assoc..

[36] Flyvbjerg A (2017). The role of the complement system in diabetic nephropathy. Nat. reviews. Nephrology.

[37] Fornoni A, Ijaz A, Tejada T, Lenz O (2008). Role of inflammation in diabetic nephropathy. Curr. Diabetes Rev..

[38] Sharma K (2005). Two-dimensional fluorescence difference gel electrophoresis analysis of the urine proteome in human diabetic nephropathy. Proteom..

[39] Sun Z, Yang P (2004). Role of imbalance between neutrophil elastase and alpha 1-antitrypsin in cancer development and progression. Lancet. Oncol..

[40] Hunt JM, Tuder R (2012). Alpha 1 anti-trypsin: one protein, many functions. Curr. Mol. Med..

[41] Rao PV (2007). Proteomic identification of urinary biomarkers of diabetic nephropathy. Diabetes care.

[42] Jin J (2012). Differential proteome profiling using iTRAQ in microalbuminuric and normoalbuminuric type 2 diabetic patients. Exp. diabetes Res..

[43] Schanstra JP (2015). Diagnosis and Prediction of CKD Progression by Assessment of Urinary Peptides. J. Am. Soc. Nephrology: JASN.

[44] Woroniecka KI (2011). Transcriptome analysis of human diabetic kidney disease. Diabetes.

[45] Tesch G (2010). Successes achieved and challenges ahead in translating biomarkers into clinical applications. AAPS J..

[46] McCarty SM, Percival SL (2013). Proteases and Delayed Wound Healing. Adv. Wound Care.

[47] Klein J, Bascands JL, Mischak H, Schanstra JP (2016). The role of urinary peptidomics in kidney disease research. Kidney Int..

[48] Lu, P., Takai, K., Weaver, V. M. & Werb, Z. Extracellular matrix degradation and remodeling in development and disease. *Cold Spring Harb Perspect Biol* **3**, 10.1101/cshperspect.a005058 (2011). 10.1101/cshperspect.a005058 PMC3225943 21917992

[49] Tan RJ, Liu Y (2012). Matrix metalloproteinases in kidney homeostasis and diseases. Am. J. Physiol. Ren. Physiol.

[50] Thrailkill KM, Clay Bunn R, Fowlkes JL (2009). Matrix metalloproteinases: their potential role in the pathogenesis of diabetic nephropathy. Endocr..

[51] Hu, X. & Beeton, C. Detection of functional matrix metalloproteinases by zymography. *J. Vis. Exp.*, 10.3791/2445 (2010). 10.3791/2445 PMC3159606 21085107

[52] Mandache E, Gherghiceanu M, Serafinceanu C, Penescu M, Mircescu G (2011). Myofibroblast involvement in tubular basement membrane remodeling in type II diabetic nephropathy. Rom. J. Morphol. Embryol..

[53] Zhao CF, Herrington DM (2016). The function of cathepsins B, D, and X in atherosclerosis. Am. J. Cardiovasc. Dis..

[54] Fox C (2016). Inhibition of lysosomal protease cathepsin D reduces renal fibrosis in murine chronic kidney disease. Sci. Rep..

[55] Brondani LA (2012). The UCP1 -3826A/G polymorphism is associated with diabetic retinopathy and increased UCP1 and MnSOD2 gene expression in human retina. Invest. Ophthalmol. Vis. Sci..

[56] Incerti J, Zelmanovitz T, Camargo JL, Gross JL, de Azevedo MJ (2005). Evaluation of tests for microalbuminuria screening in patients with diabetes. Nephrology, dialysis, transplantation: Off. Publ. Eur. Dialysis Transpl. Assoc. - Eur. Ren. Assoc..

[57] Zelmanovitz T (1997). The receiver operating characteristics curve in the evaluation of a random urine specimen as a screening test for diabetic nephropathy. Diabetes care.

[58] Paes Leme AF (2012). Hemorrhagic activity of HF3, a snake venom metalloproteinase: insights from the proteomic analysis of mouse skin and blood plasma. J. proteome Res..

[59] Broker ME (2013). Collagen peptides in urine: a new promising biomarker for the detection of colorectal liver metastases. PLoS one.

[60] Keller A, Nesvizhskii AI, Kolker E, Aebersold R (2002). Empirical statistical model to estimate the accuracy of peptide identifications made by MS/MS and database search. Anal. Chem..

[61] Nesvizhskii AI, Keller A, Kolker E, Aebersold R (2003). A statistical model for identifying proteins by tandem mass spectrometry. Anal. Chem..

[62] Zhang S, Cao X, He Y, Hartson S, Jiang H (2014). Semi-quantitative analysis of changes in the plasma peptidome of Manduca sexta larvae and their correlation with the transcriptome variations upon immune challenge. Insect Biochem. Mol. Biol..

[63] Breiman L (2001). Random Forests. Mach. Learn..

[64] Chen X, Ishwaran H (2012). Random forests for genomic data analysis. Genomics.

