# Supplementary figures and images for: Urinary peptidomics and bioinformatics for the detection of diabetic kidney disease
Source: Sci Rep. 2020 Jan 27;10:1242. doi: 10.1038/s41598-020-58067-7 (PMC6985249; doi:10.1038/s41598-020-58067-7)

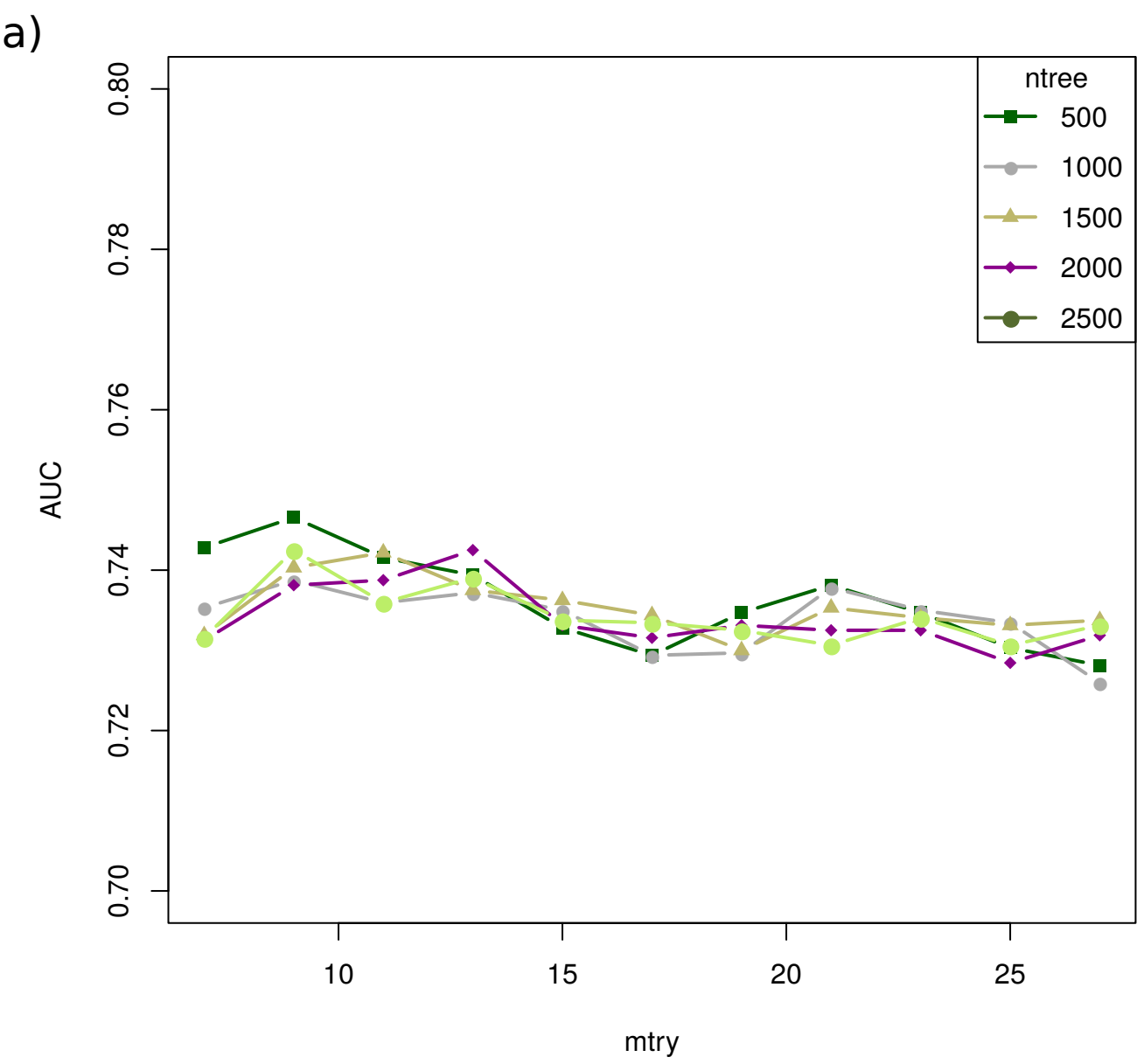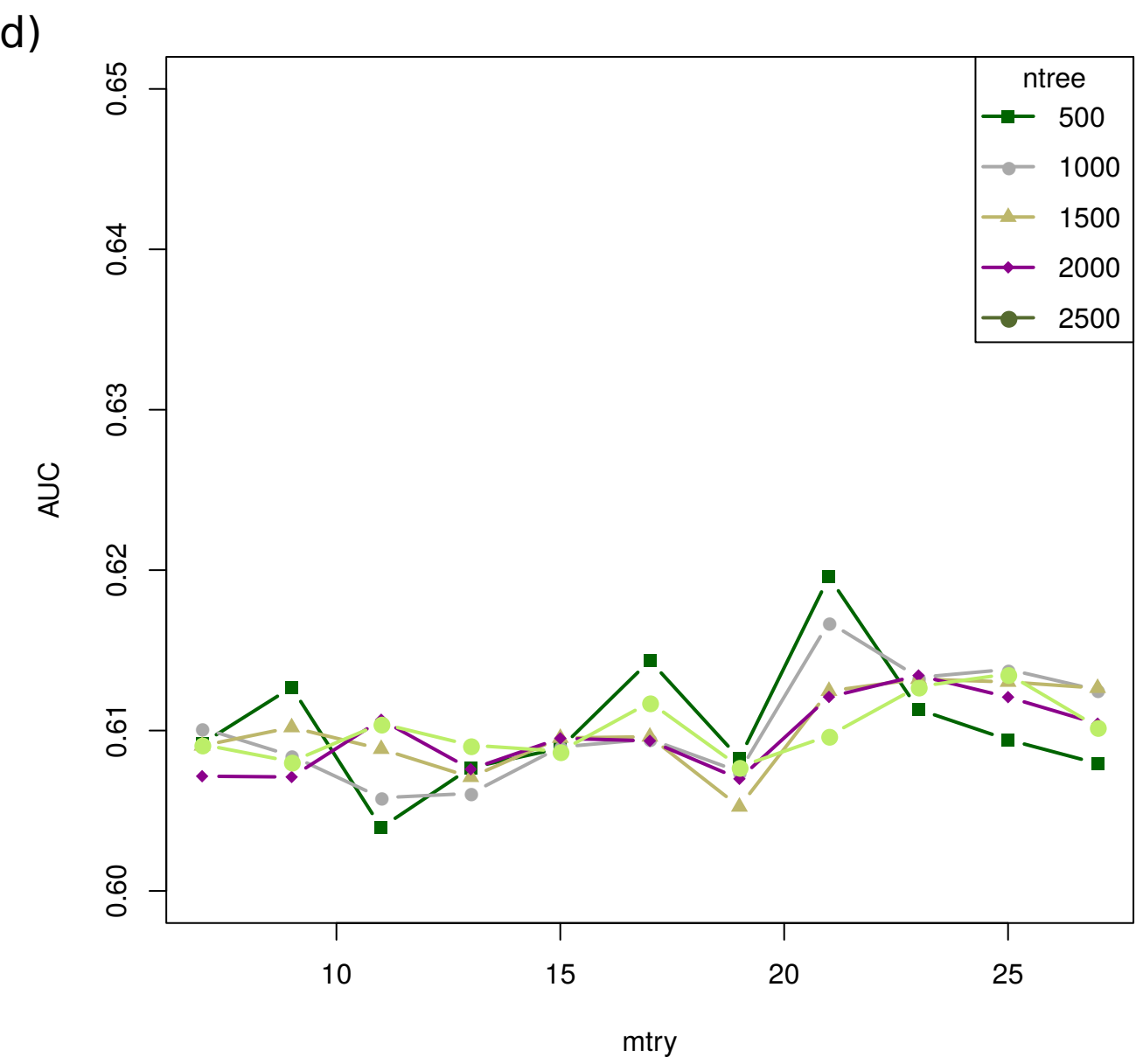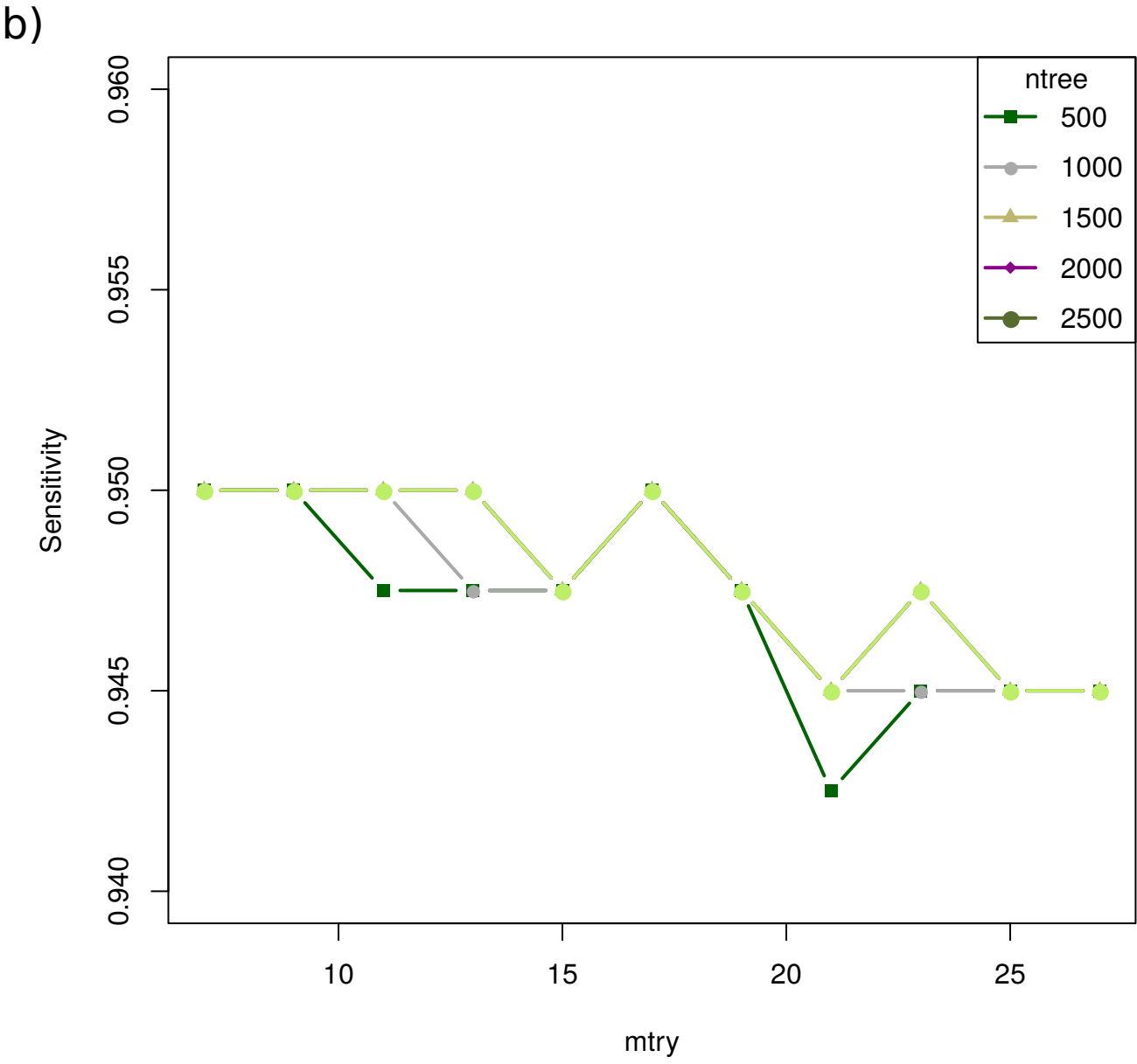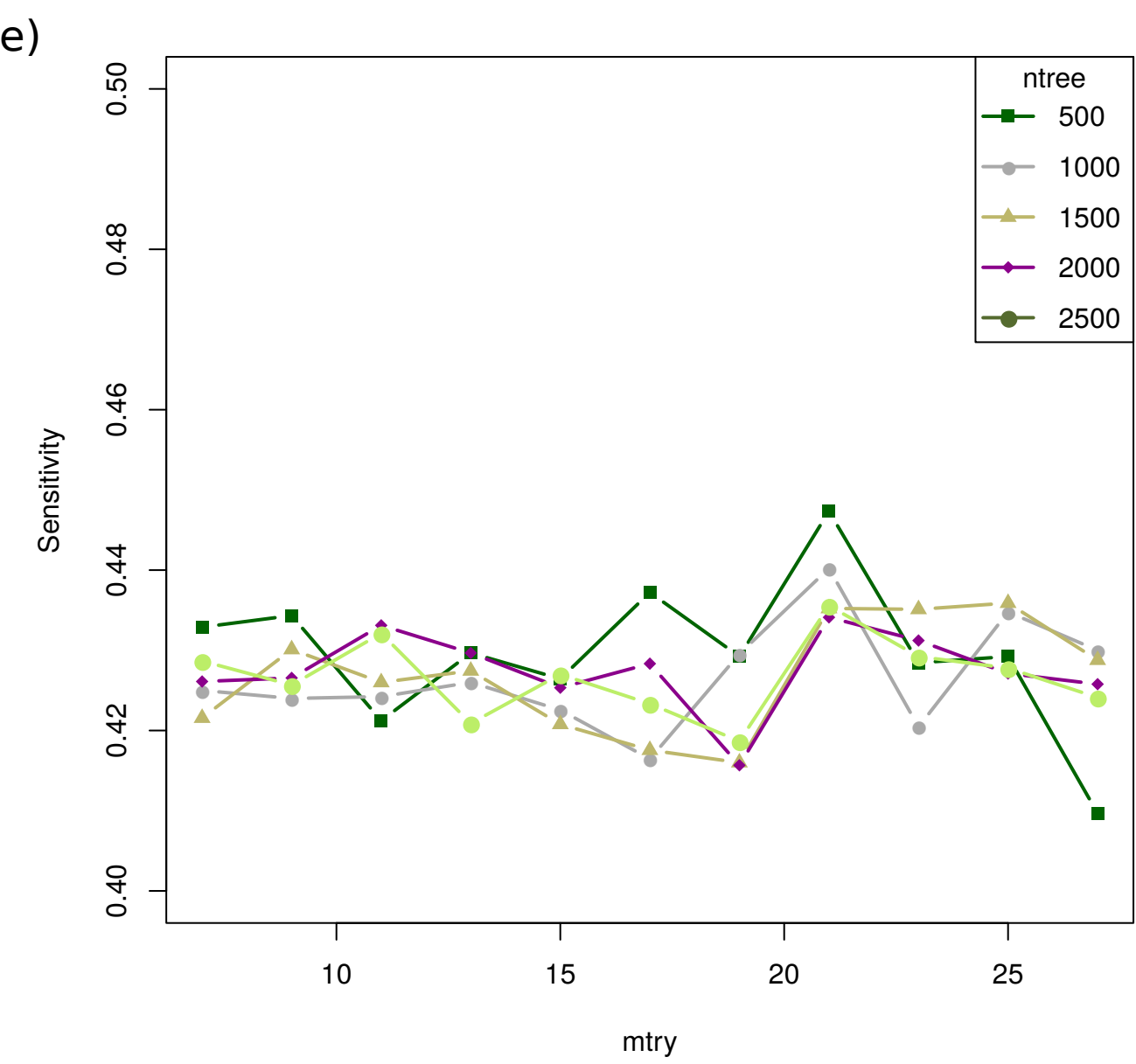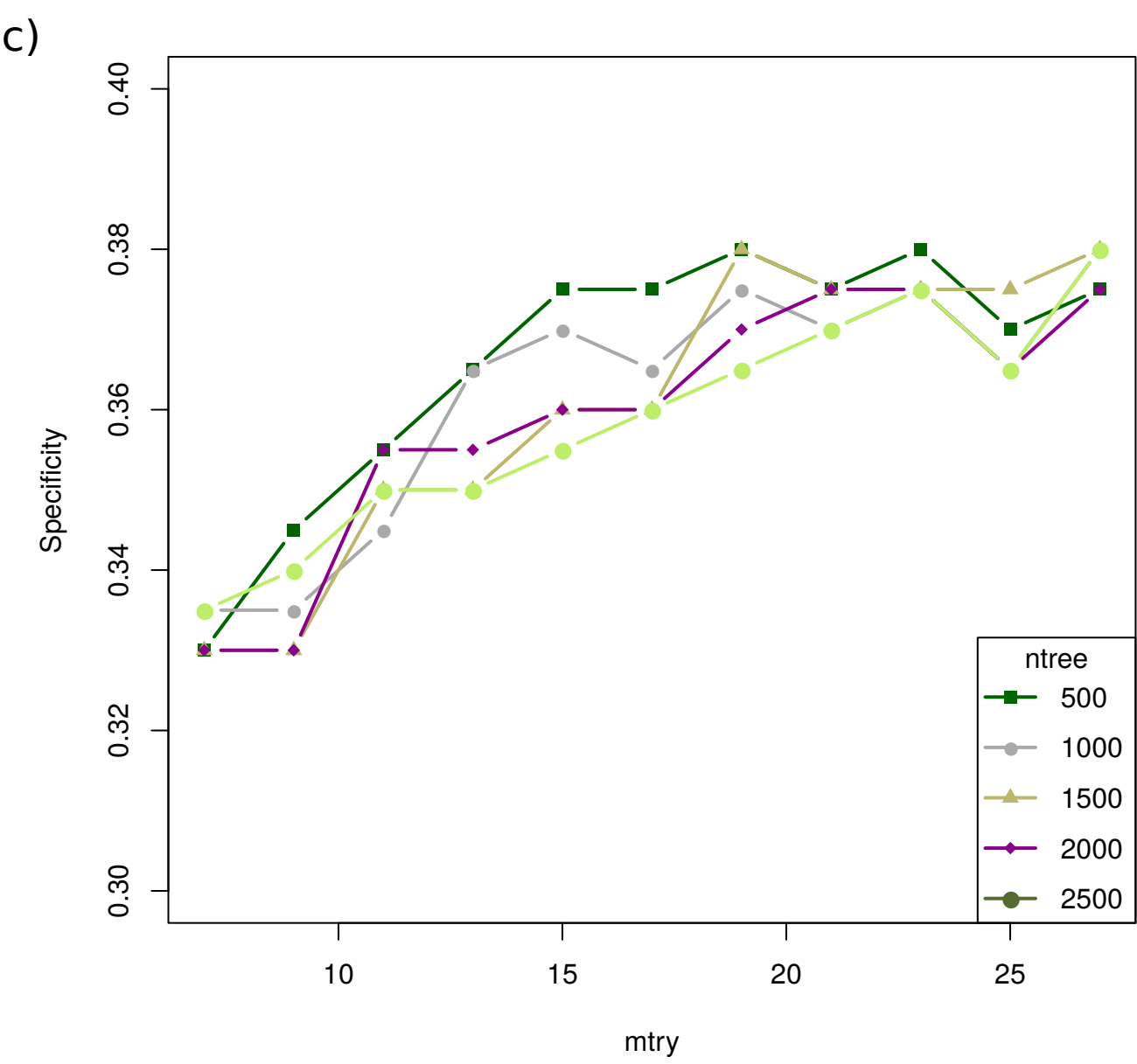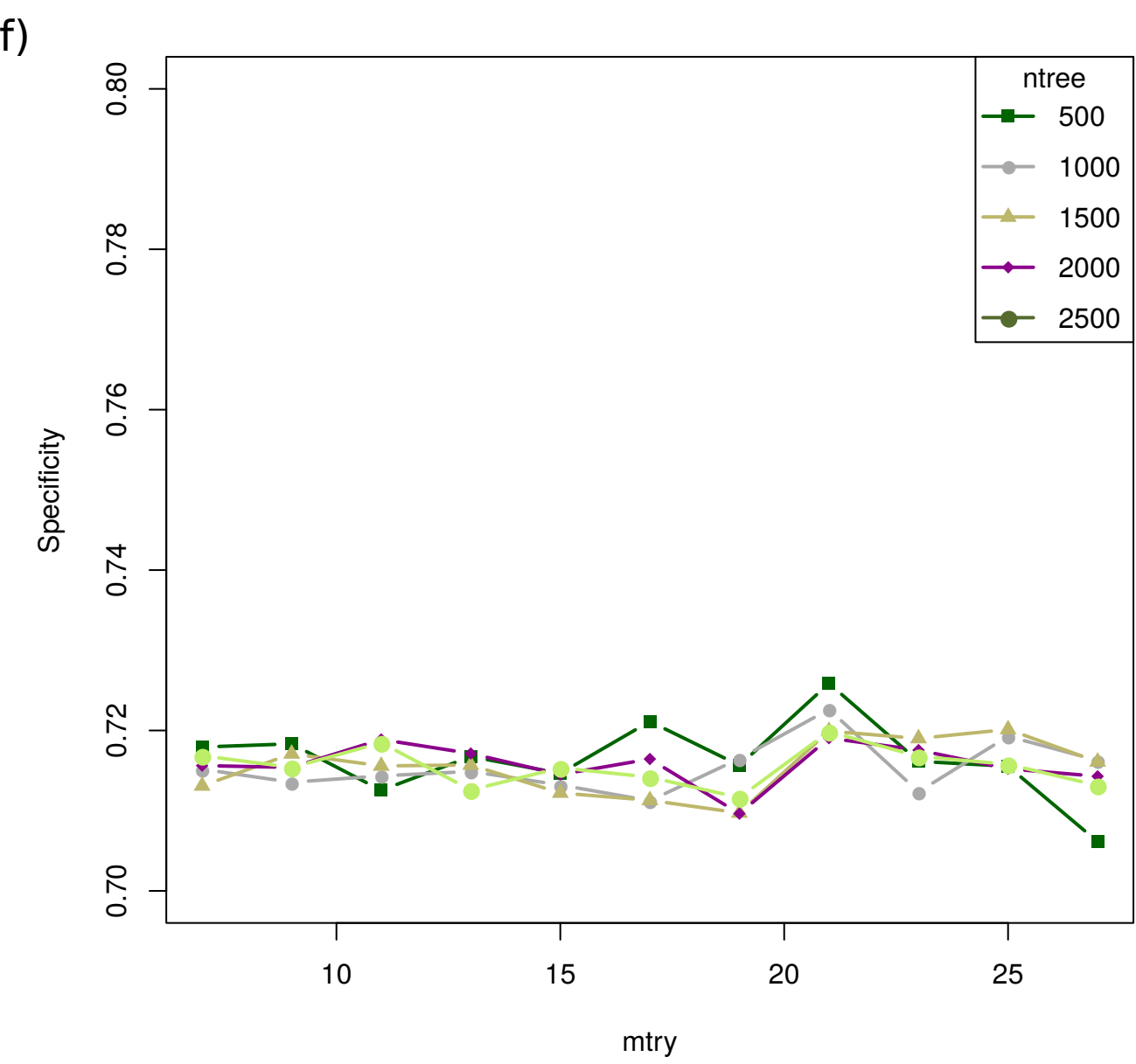

Supplement: Supplementary file 2 — Supplementary information2. [file 41598_2020_58067_MOESM2_ESM.pdf]

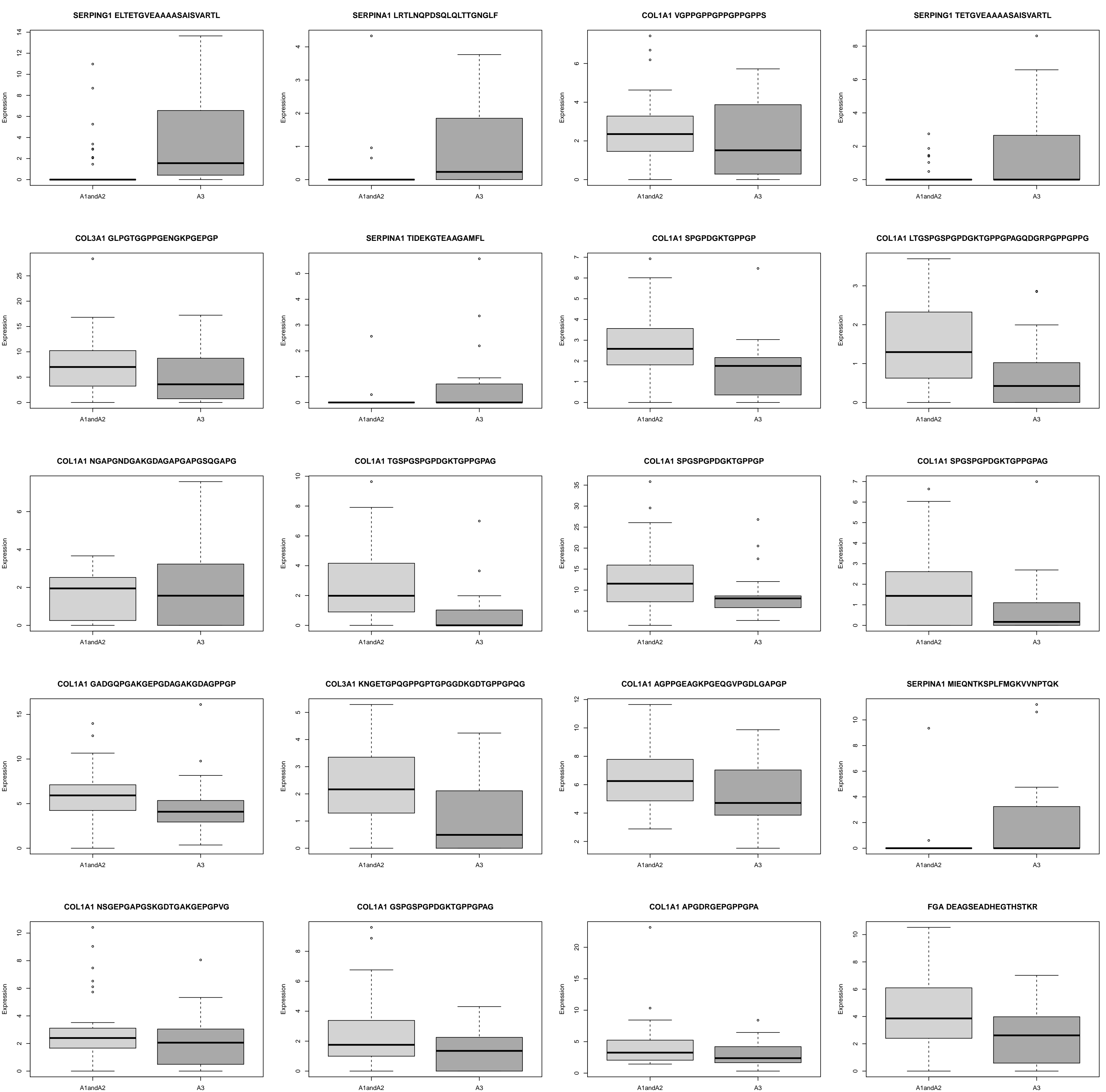

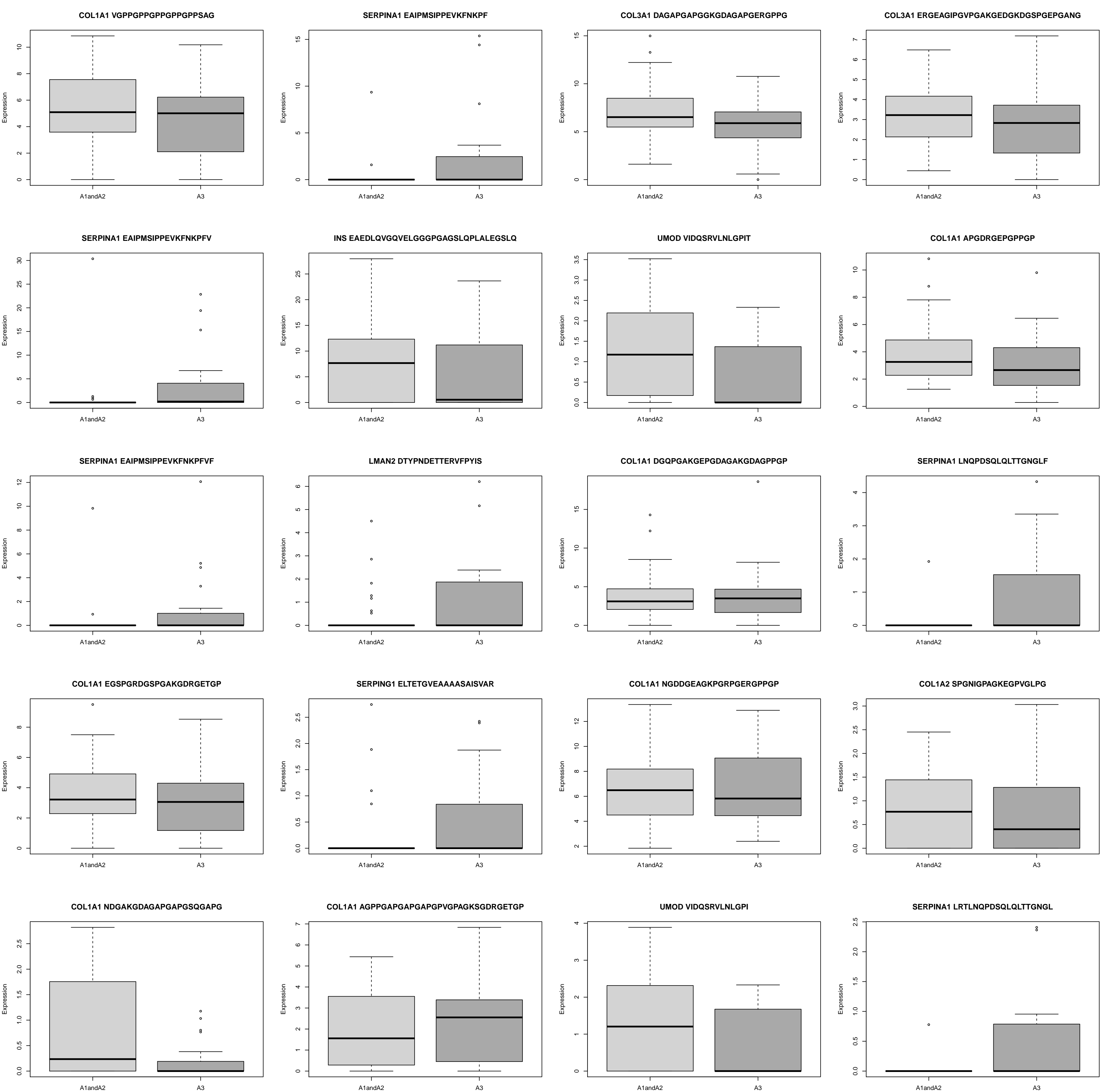

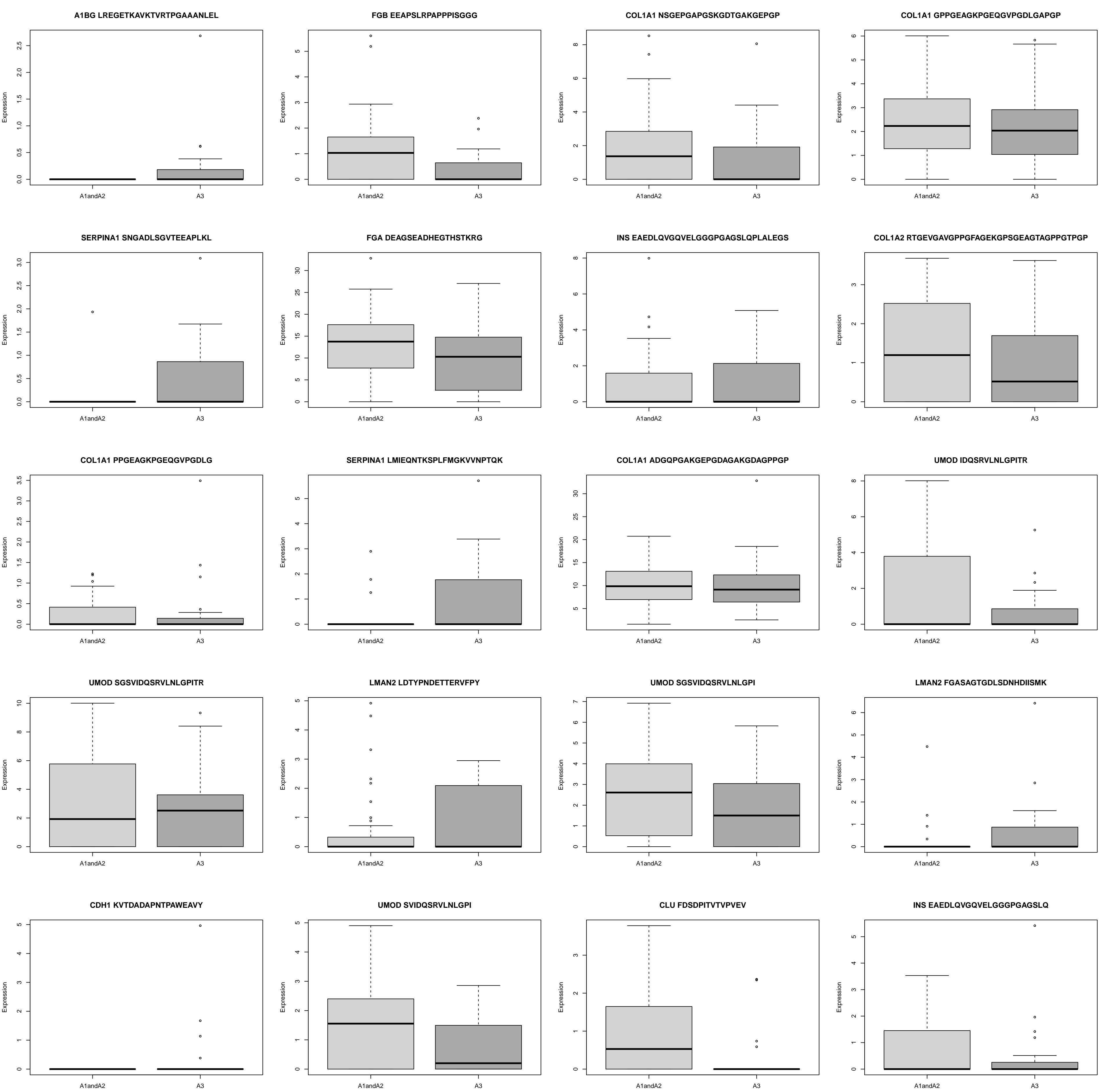

Supplement: Supplementary file 3 — Supplementary information3. [file 41598_2020_58067_MOESM3_ESM.pdf]
